# Effects of 60 Min Electrostimulation With the EXOPULSE Mollii Suit on Objective Signs of Spasticity

**DOI:** 10.3389/fneur.2021.706610

**Published:** 2021-10-15

**Authors:** Gaia Valentina Pennati, Hanna Bergling, Loïc Carment, Jörgen Borg, Påvel G. Lindberg, Susanne Palmcrantz

**Affiliations:** ^1^Karolinska Institutet, Department of Clinical Sciences, Danderyd Hospital, Division of Rehabilitation Medicine, Stockholm, Sweden; ^2^Institut de Psychiatrie et Neurosciences de Paris, Inserm U1266, Université de Paris, Paris, France

**Keywords:** stroke, muscle spasticity, electrical stimulation, medical instrument, outcome assessment

## Abstract

**Background:** The EXOPULSE Mollii method is an innovative full-body suit approach for non-invasive electrical stimulation, primarily designed to reduce disabling spasticity and improve motor function through the mechanism of reciprocal inhibition. This study aimed to evaluate the effectiveness of one session of stimulation with the EXOPULSE Mollii suit at different stimulation frequencies on objective signs of spasticity and clinical measures, and the subjective perceptions of the intervention.

**Methods:** Twenty patients in the chronic phase after stroke were enrolled in a cross-over, double-blind controlled study. Electrical stimulation delivered through EXOPULSE Mollii was applied for 60 min at two active frequencies (20 and 30 Hz) and in OFF-settings (placebo) in a randomized order, every second day. Spasticity was assessed with controlled-velocity passive muscle stretches using the NeuroFlexor hand and foot modules. Surface electromyography (EMG) for characterizing flexor carpi radialis, medial gastrocnemius, and soleus muscles activation, Modified Ashworth Scale and range of motion were used as complementary tests. Finally, a questionnaire was used to assess the participants' perceptions of using the EXOPULSE Mollii suit.

**Results:** At group level, analyses showed no significant effect of stimulation at any frequency on NeuroFlexor neural component (NC) and EMG amplitude in the upper or lower extremities (*p* > 0.35). Nevertheless, the effect was highly variable at the individual level, with eight patients exhibiting reduced NC (>1 N) in the upper extremity after stimulation at 30 Hz, 5 at 20 Hz and 3 in OFF settings. All these patients presented severe spasticity at baseline, i.e., NC > 8 N. Modified Ashworth ratings of spasticity and range of motion did not change significantly after stimulation at any frequency. Finally, 75% of participants reported an overall feeling of well-being during stimulation, with 25% patients describing a muscle-relaxing effect on the affected hand and/or foot at both 20 and 30 Hz.

**Conclusions:** The 60 min of electrical stimulation with EXOPULSE Mollii suit did not reduce spasticity consistently in the upper and lower extremities in the chronic phase after stroke. Findings suggest a need for further studies in patients with severe spasticity after stroke including repeated stimulation sessions.

**Clinical Trial Registration:** https://clinicaltrials.gov/ct2/show/NCT04076878, identifier: NCT04076878.

## Introduction

Electrical stimulation by the use of surface electrodes is a non-invasive therapeutic method used in patients with lesions of the central nervous system to improve voluntary motor control by increasing muscle strength and passive joint range of motion as well as by reducing pain and spasticity (1). Spasticity is a common manifestation of “muscle overactivity” after stroke and may negatively impact the functional recovery of patients (2–5). According to Lance's widely used definition (6), spasticity is a “motor disorder” characterized by “a velocity-dependent increase in tonic stretch-reflexes” and “resulting from hyperexcitability of the tonic stretch reflex.” Several systematic reviews conducted in the last 10 years (1, 7–10) support the effectiveness of the electrical stimulation to improve spasticity-related outcome measures across the ICF domains (11) of the body structure and function (e.g., clinical Modified Ashworth Scale) as well as activity (e.g., walking and moving), especially if used as an adjunct to the conventional therapy, like physiotherapy and botulinum toxin injection. Nevertheless, accurate recommendations of the stimulation parameters that may reduce spasticity are lacking (12, 13). For instance, the most frequently used stimulation protocol involves a high-frequency setting around 100 Hz (9, 13), although low frequencies have been applied and showed promise for reducing spasticity (14).

**Figure 1:**
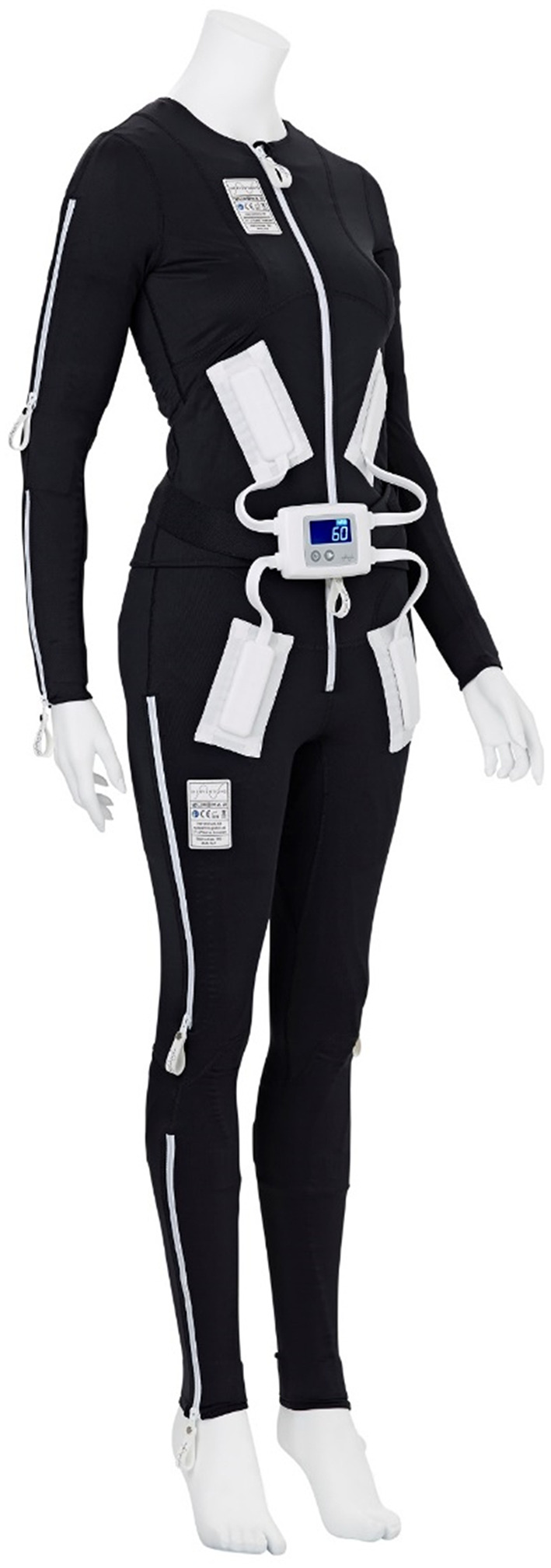
The EXOPULSE Mollii suit. This figure has been reproduced with permission from EXONEURAL NETWORK AB.

The EXOPULSE MolliiⓇ method (EXONEURAL NETWORK AB, Danderyd, Sweden) is an innovative approach for non-invasive and self-administered electrical stimulation with multiple electrodes incorporated in a full-body suit (Figure 1). It has been primarily designed to reduce disabling spasticity and improve motor function. The method refers to the concept of reciprocal inhibition elicited by stimulating the antagonist of a spastic muscle, at low frequencies and low intensities. The stimulation is considered to reduce spasticity in, e.g., an elbow flexor muscle by stimulating afferent nerve fibers of the opposing elbow extensor muscle that activates inhibitory Ia interneurons in the spinal cord and thus reducing the excitability of the flexor muscle motor neuron (15, 16). Other mechanisms of action of the EXOPULSE Mollii suit, similarly to the transcutaneous electrical nerve stimulation (TENS), may include neuroplastic changes in brain or spinal cord circuitries (10). More specifically, spasticity reduction is hypothesized to be mediated by the activation of large diameter sensory nerve afferents modulating abnormal interneuron activities in several spinal segments; by insensitivity to prolonged central excitation due to a continuous somatosensory stimulation, accompanied by lower corticomotor neuron excitability; or by synaptic reorganization of sensory and motor cortices (9).

The EXOPULSE Mollii method has already been introduced in clinical practice, and results from pilot studies in patients with stroke or cerebral palsy indicate potential benefits of this treatment on spasticity (17, 18). In a recent explorative study (19), we observed that repeated applications of EXOPULSE Mollii with sessions of 60 min every second day, at 20 Hz of stimulation, in the long-term stage after stroke may be feasible and have positive effects on spasticity and perceived functioning. However, there is a lack of studies concerning the effects of a single session of electrical stimulation with EXOPULSE Mollii on physiological measures of spasticity, as well as of studies that compare different stimulation frequencies, limiting the understanding of optimal EXOPULSE Mollii parameters.

The current study aimed to investigate the instant effects of 60 min of treatment with the EXOPULSE Mollii suit at two active frequencies (20 and 30 Hz) and in OFF-settings (placebo) with regard to objective signs of spasticity, measured with the NeuroFlexor instrument, and surface electromyography (EMG), and clinical measures. Second, the study explored the subjective perception of the treatment during each hour of stimulation with the EXOPULSE Mollii.

## Materials and Methods

### Participants

Patients >12 months after stroke, verified by CT or MRI examination, were recruited in a double-blind controlled, randomized, cross-over study.

Inclusion criteria were: >17 years of age; presence of spasticity according to clinical assessment; hemiplegia, including both upper and lower extremity; limited ability to walk (scores 2–4 according to the Functional Ambulatory Categories combined with a gait speed <0.8 m/s during the 10-m walk test); limited activity in the upper extremity according to the Action Research Arm test with at least partial voluntary ability to grasp, grip, and perform gross arm movement (i.e., score >1 point on each of these items); ability to understand oral and written information.

Exclusion criteria comprised no objective sign of spasticity according to NeuroFlexor measure, i.e., neural component in the upper extremity <3.4 N (20); contractures limiting passive range of movement required for NeuroFlexor assessment; any other neurological or orthopedic disorder with an impact on sensorimotor function; any other severe concomitant disease (such as cancer, cardiovascular, inflammatory, or psychiatric disease); uncontrolled epilepsy or blood pressure; major surgery during the last year; any implanted medical devices; pregnancy; and BMI > 35 (available EXOPULSE Mollii suit sizes: XS–XXXL).

Participants with ongoing pharmacological treatment for spasticity could be included in the study only if oral anti-spastic medication was stable since at least 3 months, and no change during the study period was anticipated. As well, participants who received intramuscular injection for spasticity could participate only if the time since last treatment was at least 3 months, and no new injection would be given during the study period.

### Intervention Protocol

The EXOPULSE Mollii suit consists of tight-fitting jacket and trousers incorporating 58 electrodes. Only a subset of the electrodes was activated for each participant in order to apply the electrical stimulus to the antagonists of spastic muscles (including the extensor muscles of the hand and the anterior tibial muscle), based upon the initial clinical assessment of the patient performed by a physiotherapist (as presented below in Data collection and Outcomes). In addition, abdominal and back muscles were stimulated to follow the methodology for setting the EXOPULSE Mollii suit, previously developed and adopted by therapists in clinical practice.

Each patient underwent a total of three intervention sessions every second day, each of which lasted for 60 min and with a different stimulation frequency. In addition to the active stimulations at 20 Hz (the standardized frequency based on clinical experience) and 30 Hz (the maximum frequency anticipated by the suit), OFF- settings was used for placebo stimulation. The order in which the frequencies were tested on the patients was randomized and double-blinded.

The EXOPULSE Mollii Suit generates 2-mA pulses with the following parameters: pulse width, personalized upon the initial clinical evaluation of the patient and variable between 40 and 90 μs for muscles of the upper limb and between 45 and 120 μs for muscles of the lower limb (including pulse width 50–90 μs for the extensor carpi ulnaris and 65–120 μs for the anterior tibial muscle); pulse shape, square wave; maximum amplitude, 20 V. The pulse width remained the same for both the intervention sessions with active stimulation. Stimulus intensity was also individualized and took into account the estimated severity of spasticity, size of the muscle at focus, if it was weight bearing or not, and the body composition of the patient.

The stimulation parameters were set based on clinical experience, by a member of the manufacturer (EXONEURAL NETWORK AB) who was not otherwise involved in the study. Moreover, the participants were informed that the electrical stimulation delivered through EXOPULSE Mollii may be perceived as a transient and slight tingling sensation in some people, or not at all.

Please view Supplementary Figure 1 for more details.

### Data Collection and Outcomes

A physiotherapist and a medical doctor blinded to the frequency of stimulation conducted the assessments before, during, and after each intervention session at day 1, 2, and 3.

Data regarding age, gender, independence in walking [using the Functional Ambulation Categories (FAC) (21), ranging from 0 = non-functional ambulation to 5 = independent ambulation], and independence in mobility and personal care [using the Barthel Index (22), ranging from 0 = dependence in mobility and personal care, to 100 = independence] were collected. In addition, information about stroke type (ischemia or hemorrhage), time to inclusion from stroke onset and paretic side were obtained. Sensorimotor function including voluntary and passive movement, tactile sensibility to light touch and proprioception and pain were rated according to the Fugl–Meyer scale for upper (FMA–UE) and lower extremity (FMA–LE), yielding a maximum of 126 and 86 points, respectively, where a lower score indicates more severe impairment (23).

#### Measure of Spasticity

Changes in spasticity during and after the use of the EXOPULSE Mollii suit represented the main outcome and were investigated with the NeuroFlexor hand and foot modules. Additionally, surface electromyography (EMG) was used to evaluate reflex muscle activity in the spastic muscles in the upper and lower limb.

NeuroFlexor^TM^ (Aggero MedTech AB, Älta, Sweden) is a validated method (24, 25) that quantifies the neural component (NC) reflecting stretch reflex-mediated resistance, i.e., spasticity, and the mechanical contributions (elastic and viscous components, EC, and VC) of the total resistance opposing a passive stretch. More specifically, the NeuroFlexor instrument performs passive movements at controlled velocities, and the incorporated biomechanical algorithm estimates the active contractions evoked by stretch reflexes (NC) by subtracting the length-dependent resisting force (EC) and the force due to friction of sliding muscle fibers (VC) from the total resistance at the maximal degree of angular displacement. Please view Supplementary Material for more details. The assessment procedure was similar to that used in previous studies (20, 24–26). Briefly, the participants were seated on a height-adjustable chair in a comfortable seating position with the hand and the foot placed on the platform of the NeuroFlexor device. The hand and the foot were tested separately, starting with the upper extremity. The participants were instructed to relax during the testing session, which consisted of passive extension of the wrist and dorsiflexion of the ankle at two different isokinetic velocities, slow (5°/s) and fast (236°/s for the upper extremity and 240°/s for the lower extremity). The total range of wrist movement was 50° (range −20°-+30°) and the total range of ankle movement was at least 30°, within the range of 35° of plantarflexion to 5° of dorsiflexion (or −5° in case of limited range of motion). The knee joint was positioned at a flexion angle of 45°. For each patient, one value of NC, EC, VC, and total resistance was calculated in a dedicated software (NeuroFlexor Scientific, Release 1.0.0) using the mean of the NeuroFlexor components obtained from the latest four slow and nine fast passive movements (the first movements of both velocities were excluded to limit contamination by startle response).

Surface EMG of the flexor carpi radialis, gastrocnemius, and soleus muscles was recorded synchronized with the NeuroFlexor assessment, before and after stimulation. Disposable Ag/AgCl surface electrodes were placed in a belly-tendon montage aligned with the muscle fibers using the SENIAM electrode placement guidelines (27). EMG signal was amplified with a Grass LP511 Ac Amplifier (Grass Technologies, Astro-Med, Inc., West Warwick, RI, USA), sampled at 1 kHz using a CED Power1401 (Cambridge Electronic Designs, Cambridge, UK) and rectified. The root mean square of the EMG signal, with 50 ms sliding window, was computed to generate the EMG amplitude during the whole NeuroFlexor passive movement from onset until full extension of wrist and ankle dorsiflexion. Data were acquired with Spike2 software (Version 7.12; CED) and analyzed off-line using custom-written programs in MATLAB R2017b (The MathWorks, Inc., Natick, Massachusetts, USA).

In addition, the Modified Ashworth scale (MAS), a five-point ordinal scale (from 0 = no spasticity, to 4 = fixed muscle contracture), was used to assess clinical ratings of spasticity. The scale does not discriminate spasticity from other factors contributing to passive movement resistance, but it is widely applied in clinical practice as well as in clinical research and was therefore used to describe the study sample and for further comparison with other studies. Finally, the presence of clonus elicited during MAS assessment was recorded as no clonus, mild = 1–3 beats, moderate = 4–10 beats, and sustained.

#### Additional Clinical Measure

Although spasticity is neural in origin, concomitant changes in the soft tissue occur generally after stroke. Thus, limitation in joint movement is an important component of evaluation. Passive and active range of motion (ROM) of wrist and ankle were measured by the use of a goniometer before and after stimulation, and a change in ROM measurements >5° was considered clinically significant (28).

#### Subjective Perceptions of the Exopulse Mollii

**Figure 2:**
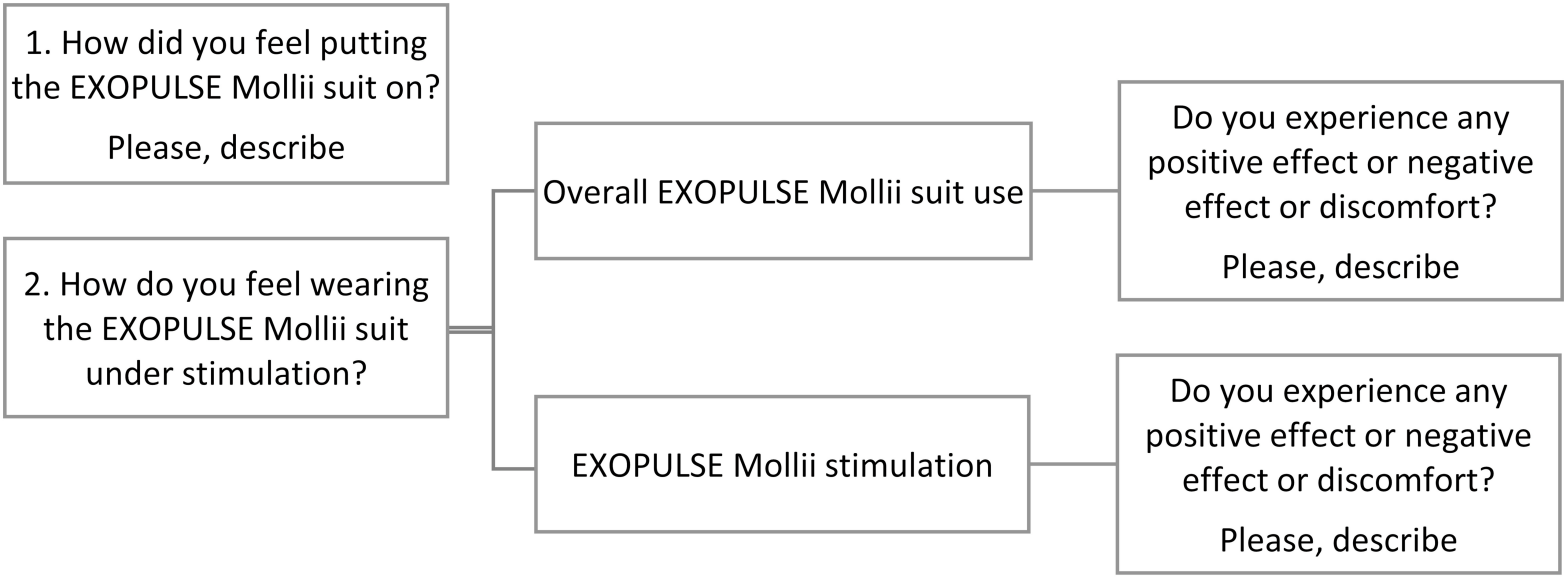
Semi-structured questions asked to the patients during each of the three sessions of stimulation with EXOPULSE Mollii.

Participants were interviewed regarding their perceived effects of EXOPULSE Mollii during each hour of stimulation by an experienced blinded physiotherapist. A standardized questionnaire was adopted. The interviewer read the questions (presented in Figure 2), and the participants were asked to provide oral answers in their own words. Responses of patients, collected by the interviewer were grouped for further analysis, and the number of participants reporting in each group was calculated.

The patients were also asked to rate the effort of putting the suit on and the potential discomfort during the stimulation on a 10-point scale ranging from 1 (no effort/no discomfort) to 10 (maximum value of effort/discomfort).

### Statistical Analysis

Descriptive statistics, expressed as mean ± standard deviation (SD) or median [interquartile range (IQR)] for not normally distributed data (detected with the Shapiro–Wilk test), was used to characterize the study population.

After natural log transformation (applied to correct skewed distribution), a repeated measures analysis of variance (rm-ANOVA) investigated the difference in NC and NeuroFlexor total resistance quantified before stimulation between days 1, 2, and 3 in order to evaluate the potential fluctuations in spasticity over the course of the study.

Two-way repeated measures ANOVAs were conducted to examine the effects of stimulation delivered through EXOPULSE Mollii and the different stimulation frequencies (OFF settings, 20 and 30 Hz) on log transformed NC and EMG amplitude, MAS, and active and passive ROM.

Spearman's rank order correlation test (*r*_*s*_) was used to investigate relations between original NC and EMG data (since *r*_*s*_ of raw data was stronger than Pearson's correlation coefficient of transformed data).

At individual level, changes in spasticity were explored by calculating the difference in NC between after stimulation value and before stimulation value (Delta_A−B_ NC), and are presented graphically. A variation of NC higher than 1 N [i.e., beyond the margin of measurement error of the biomechanical model (24)] was considered a true value.

The level of statistical significance was set at *p* ≤ 0.05. All statistical analyses were performed using IBM SPSS Statistics for Windows, Version 26.0 (Armonk, NY: IBM Corp).

## Results

### Characteristics of Participants

Twenty patients were evaluated with the NeuroFlexor hand module and the EMG in the forearm flexor. A sub-group of 10 patients were properly evaluated with the NeuroFlexor foot module and the EMG in the calf muscles. This group was smaller in size as excessive resistance to the passive muscle stretch triggered the preventive safety stop of the NeuroFlexor foot module in two patients, while the data of the remaining eight participants were excluded from the analyses due to violation of the NeuroFlexor assessment protocol (i.e., different range of ankle movement and knee angle, or severe malposition of the foot on the NeuroFlexor platform due to the difficulty for these participants to adopt the standardized positions).

Characteristics of the included participants are shown in Table 1. FMA–UE showed moderate-severe upper limb motor impairment, with only three participants out of 20 with intact tactile sensory function (mean 2.50 ± SD 1.61) and proprioception (3.45 ± 3.07). According to FMA–LE, participants presented a moderate motor impairment (19.60 ± 5.68), with only two patients with intact tactile sensory function (2.75 ± 1.26) and proprioception (4.35 ± 2.59) in the lower limb.

**Table 1:** Demographic data and patient characteristics.

**Variables**	***N*** **= 20**
**Age**, mean ± SD, range	58.80 ± 13.11, 28–79
**Sex**, *n* (%)	
Female	7 (35)
Male	13 (65)
**Months from stroke onset to inclusion**, mean ± SD, range	62.15 ± 44.09, 16–172
**Stroke type**, n (%)	
Ischemic	11 (55)
Hemorrhagic	9 (45)
**Paretic side**, *n* (%)	
Left	10 (50)
Right	10 (50)
**Independence in walking, FAC**, (0–5 p)^*^, median (IQR), range	4.50 (1.00), 2–5
**Self-care and mobility, Barthel Index**, (0–100 p)^*^, median (IQR), range	95.00 (15.00), 40–100
**FMA–UE**, (0–126 p)^*^, mean ± SD, range	77.10 ± 17.92, 53–117
**FMA–UE, motor score**, (0–66 p)^*^, mean ± SD, range	29.80 ± 14.70, 8–59
**FMA–UE, sensory function**, (0–12 p)^*^, mean ± SD, range	5.95 ± 4.44, 0–12
**FMA–UE, joint pain** (0–24 p)^*^, median (IQR), range	24.00 (2.00), 20–24
**FMA–LE**, (0–86 p)^*^, mean ± SD, range	63.15 ± 6.94, 53–76
**FMA–LE, motor score**, (0–34 p)^*^, mean ± SD, range	19.60 ± 5.68, 9–31
**FMA–LE, sensory function**, (0–12 p)^*^, mean ± SD, range	7.10 ± 3.64, 0–12
**FMA–LE, joint pain** (0–20 p)^*^, median (IQR), range	20.00 (1.00), 18–20

* *Score range (minimum–maximum point): a lower score indicates increased impairment or limitation.*

*FAC, Functional Ambulation Categories; FMA, Fugl–Meyer assessment for upper (UE) and lower extremity (LE)*.

### Evaluation of Baseline Spasticity in the Upper Extremity Every Second Day

NC before the intervention reduced slightly from day 1, to 2, to 3 in the upper extremity [median 13.70 N (IQR 9.77), 12.59 N (10.51), and 11.35 N (14.99), respectively]. However, the rmANOVA showed no significant change of NC before stimulation across days [*F*_(2,38)_ = 0.057, *p* = 0.944]. There was also no significant difference (*p* = 0.66) between the NeuroFlexor total resistance before electrical stimulation acquired at day 1, 2, and 3 [median 17.67 N (IQR 11.88), 18.37 N (12.07), and 16.32 N (14.36), respectively].

### EXOPULSE Mollii Stimulation-Associated Changes in Spasticity in the Upper Extremity

#### NeuroFlexor Components and Electromyography Amplitude

**Figure 3:**
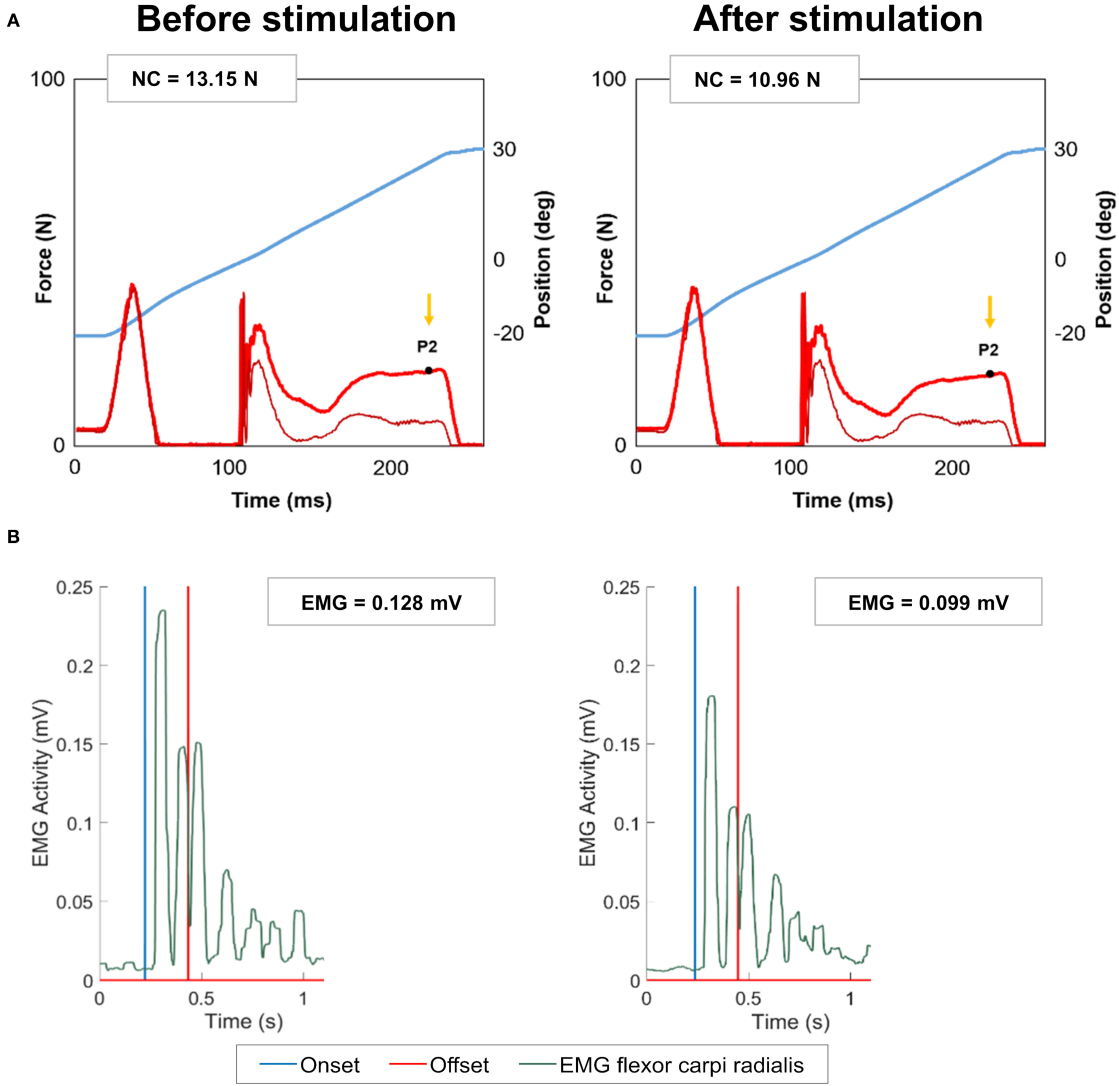
Example of NeuroFlexor force trace and electromyography signal before and after stimulation with EXOPULSE Mollii. **(A)** NeuroFlexor hand module resistance profile during the fast velocity movement, before and after 60 min of electrical stimulation with EXOPULSE Mollii at 30 Hz. Blue trace shows the angle of wrist movement, from 20° of palmar flexion to 30° of extension. Bright red trace shows resisting force to the passive stretch and the ticker dark red line shows resistance profile when the device ran without hand. Arrow shows the late resistance toward the end of the movement (P2 time point). Values of neural component (NC) are reported in Newton, N. **(B)** Electromyography (EMG) signal of the flexor carpi radialis recorded synchronized with the NeuroFlexor assessment from onset (i.e., 20° of flexion) until full extension of wrist (offset, i.e., 30°). The amplitude of EMG signal is reported in mV. After stimulation, a decreased NC was accompanied by a reduced EMG amplitude (i.e., smaller burst in EMG signal in green).

Figure 3 shows an example of NeuroFlexor hand module resistance profiles and EMG signal of the flexor carpi radialis, before and after 60 min of electrical stimulation with EXOPULSE Mollii at 30 Hz. At group level, NC and total resistance increased slightly after stimulation at all frequencies as reported in Table 2. The EMG amplitude of the flexor carpi radialis decreased moderately after stimulation at 30 and in OFF settings (a 2 and 4% decrease in median, respectively) while it increased at 20 Hz (a 22% increase in median), as presented in Table 2.

**Table 2:** NeuroFlexor components and amplitude of electromyography signal before, during, and after stimulation with EXOPULSE Mollii.

**Variables**		**OFF-settings**			**20 Hz**			**30 Hz**		
		**Before**	**During**	**After**	**Before**	**During**	**After**	**Before**	**During**	**After**
Upper extremity	NC	11.24 (11.96)	12.30 (10.80)	13.87 (7.20)	12.28 (11.68)	12.71 (10.17)	13.44 (10.78)	13.35 (8.88)	12.97 (9.05)	14.76 (12.52)
	EC	3.85 (2.80)	4.60 (3.01)	4.15 (3.25)	4.07 (3.76)	4.80 (4.63)	4.22 (2.54)	4.08 (3.33)	4.57 (3.67)	3.56 (2.40)
	Total resistance	18.27 (12.74)	17.24 (13.75)	18.44 (7.61)	16.70 (12.72)	18.50 (11.68)	18.98 (9.83)	17.17 (11.57)	19.41 (10.40)	19.31 (13.63)
	FCR EMG	0.072 (0.04)		0.069 (0.05)	0.061 (0.06)		0.075 (0.09)	0.061 (0.04)		0.060 (0.05)
Lower extremity	NC	33.64 (34.22)	31.54 (27.19)	34.02 (33.45)	38.85 (29.54)	35.16 (34.29)	31.41 (38.04)	35.31 (34.57)	33.66 (36.00)	37.54 (31.65)
	EC	59.63 (25.95)	58.04 (32.85)	63.67 (33.51)	53.14 (30.14)	60.73 (16.65)	60.14 (29.11)	59.04 (17.91)	60.93 (14.86)	60.69 (22.74)
	Total resistance	104.42 (29.75)	100.16 (37.96)	113.47 (28.00)	102.86 (26.88)	109.06 (24.79)	104.49 (26.89)	104.66 (27.22)	111.52 (32.02)	106.79 (28.05)
	Gastrocnemius EMG	0.053 (0.03)		0.059 (0.03)	0.048 (0.02)		0.053 (0.05)	0.052 (0.02)		0.048 (0.04)
	Soleus EMG	0.067 (0.05)		0.057 (0.07)	0.053 (0.05)		0.055 (0.04)	0.045 (0.03)		0.052 (0.03)

*Values in median (IQR).*

*NeuroFlexor neural component (NC), elastic component (EC), and total resistance (in Newton, N), and amplitude of electromyography (EMG) signal (in mV) in the upper extremity (N = 20) and lower extremity (n = 10). FCR, flexor carpi radialis muscle*.

**Figure 4:**
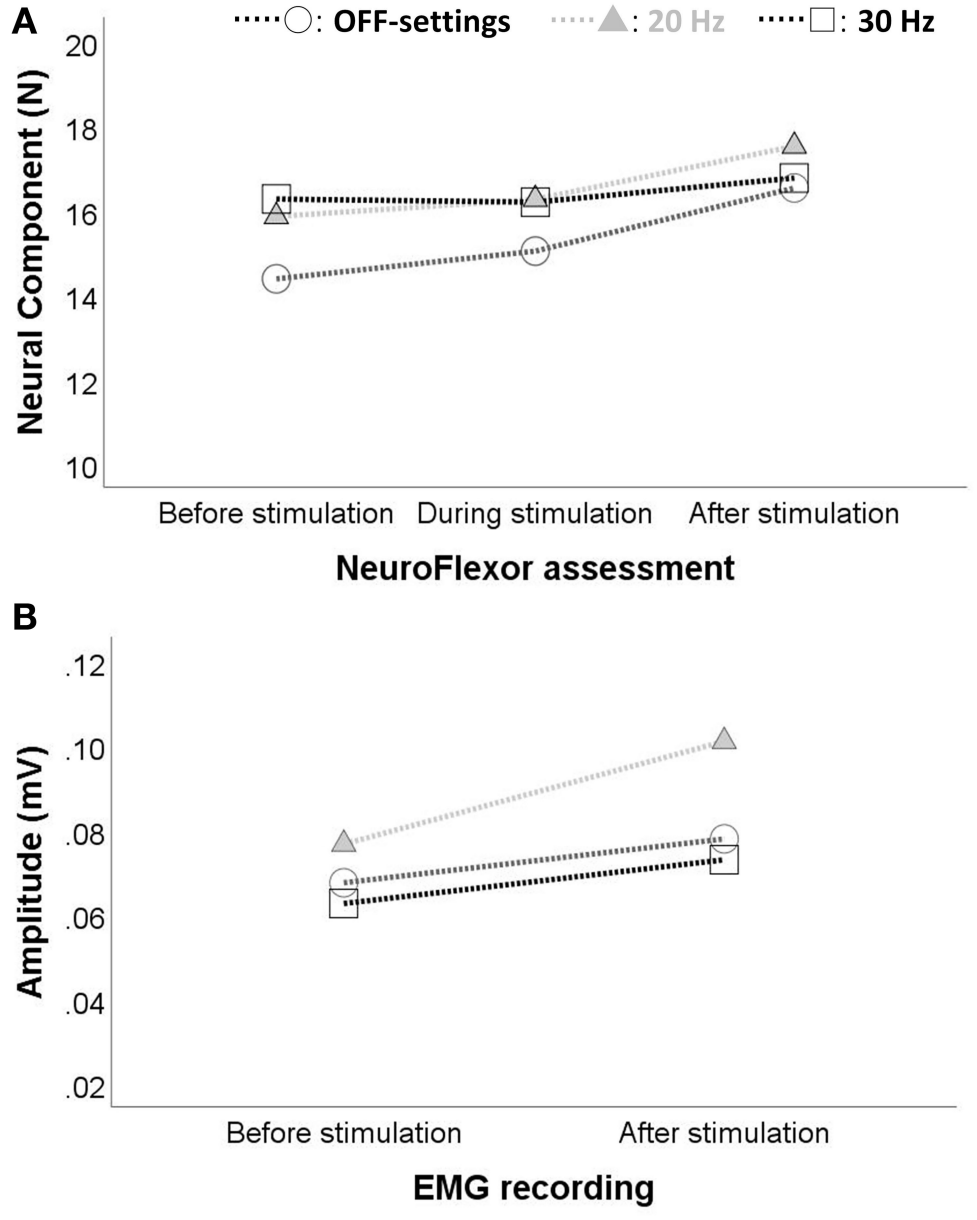
Changes in spasticity of the upper extremity during and after stimulation with EXOPULSE Mollii. A slight increase (*p* > 0.05) in **(A)** the NeuroFlexor neural component (in Newton, N) and in **(B)** the electromyography (EMG) amplitude of the flexor carpi radialis (in mV) was observed during and after stimulation with the EXOPULSE Mollii suit at different frequencies: OFF-settings = circles, 20 Hz = solid triangle and 30 Hz = square.

Two-way rmANOVAs showed no significant interaction effect of electrical stimulation and frequencies on NC [*F*_(4,76)_ = 1.043, *p* = 0.352] and EMG amplitude [*F*_(2,38)_ = 0.989, *p* = 0.381]. Nevertheless, both NC in the upper extremity and EMG amplitude of the flexor carpi radialis presented a similar slight increasing trend during and at the end of a stimulation session, as shown in Figure 4. The parallel pattern of NC and EMG amplitude was confirmed by Spearman's correlation, which revealed a significant correlation before and after stimulation at 20 Hz (*r*_*s*_ = 0.69, *p* < 0.01 and *r*_*s*_= 0.51, *p* = 0.02, respectively), and before stimulation at 30 Hz (*r*_*s*_ = 0.53, *p* = 0.02).

#### Clinical Changes in Spasticity and Joint Movement

At group level, MAS values showed no change within or across sessions (*p* > 0.40; Table 3). Moreover, mild (i.e., 1–3 beats) or moderate (i.e., 4–10 beats) clonus was elicited at wrist and fingers in 25% patients before OFF settings stimulation, 35% at 20 Hz, and 30% at 30 Hz, and remained essentially stable after 60 min of treatment.

**Table 3:** Modified Ashworth scale (MAS) before and after stimulation with EXOPULSE Mollii (*N* = 20).

**Variables**	**OFF-settings**		**20 Hz**		**30 Hz**	
	**Before**	**After**	**Before**	**After**	**Before**	**After**
**MAS, upper extremity** (0–35 p)^*^	9.50 (4.75)	9.00 (6.50)	9.00 (4.75)	8.00 (5.75)	9.00 (5.75)	9.50 (6.50)
**MAS, wrist flexors** (0–5 p)^*^	1.00 (2.00)	1.00 (2.00)	1.00 (2.00)	1.00 (2.00)	2.00 (2.75)	2.00 (2.00)
**MAS, lower extremity** (0–30 p)^*^	5.00 (5.50)	6.00 (4.00)	5.00 (5.50)	4.00 (5.50)	5.00 (5.75)	6.00 (6.25)
**MAS, gastrocnemius muscle** (0–5 p)^*^	1.00 (3.00)	0.50 (2.00)	0.50 (2.00)	0.50 (2.00)	0.50 (2.00)	1.00 (2.75)
**MAS, soleus muscle** (0–5 p)^*^	0.00 (1.00)	0.00 (4.00)	0.50 (2.00)	0.00 (2.00)	0.00 (2.00)	0.00 (2.00)

*Values in median (IQR).*

* *Score range (minimum–maximum point).*

*MAS, soleus muscle measured with the knee flexed to ~15°*.

Regarding ROM changes in upper extremity, no group effect was observed in active or passive wrist extension (without or with fingers extended) at any frequency (*p* > 0.30). At individual level, three patients out of 20 increased significantly active wrist extension without fingers extended in OFF settings (+10°), four patients at 20 Hz and three at 30 Hz (increase range +10–+20°). Two patients achieved a greater passive ROM, with an increase of +10° in OFF-settings and 20 Hz, and of +20° at 30 Hz. Again, one patient increased active wrist extension with fingers extended in OFF-settings (+15°), four patients at 20 Hz and two at 30 Hz (increase range +10–+20°). Three patients exhibited increased passive ROM with fingers extended in OFF settings and five patients at 20 and 30 Hz, with the maximum increase of +30°.

### Evaluation of Baseline Spasticity in the Lower Extremity Every Second Day

In the lower extremity of the sub sample, NC before stimulation decreased from day 1 to 2 [median 35.08 N (IQR 35.11) and 32.83 N (28.39), respectively], but increased at day 3 [37.12 N (31.69)]. As for the upper extremity, the rmANOVA determined that NC before stimulation did not differ significantly between time points in the lower limb [*F*_(2,18)_ = 0.180, *p* = 0.837]. A slight decrease of the NeuroFlexor total resistance was seen over time [109.09 N (25.65) at day 1, 102.86 N (33.55) at day 2 and 97.77 (26.66) at day 3], but this was not statistically significant (*p* = 0.31).

### EXOPULSE Mollii Stimulation-Associated Changes in Spasticity in the Lower Extremity

#### NeuroFlexor Components and Electromyography Amplitude

NC decreased during stimulation at all frequencies but increased again after stimulation in OFF-settings and 30 Hz, as presented in Table 2. This table also shows the moderate variations in amplitude of the gastrocnemius and soleus muscles activity after stimulation at different frequencies.

Two-way rmANOVAs showed no interaction or simple main effects of electrical stimulation and frequencies on NC, gastrocnemius and soleus EMG amplitude (*p* = 0.803, 0.644, and 0.625, respectively, for the three interaction effects).

As for the upper extremity, the correlation analysis showed a significant relationship between NC and EMG amplitude (soleus muscle) before stimulation in the lower limb (*r*_*s*_ = 0.81, *p* < 0.01 at 20 Hz and *r*_*s*_ = 0.79, *p* < 0.01 at 30 Hz).

#### Clinical Changes in Spasticity and Joint Movement

The clinical assessment of spasticity in the lower limb is reported in Table 3. In addition, ankle clonus elicited in gastrocnemius muscle reduced in severity after active frequencies: from four patients with moderate and three with sustained clonus before stimulation to two patients with mild clonus, two moderate and three sustained after 20 Hz of stimulation, and from one patient with mild clonus, four with moderate and three with sustained before stimulation to one patient with mild clonus, one moderate and two sustained after stimulation with 30 Hz.

Regarding ROM, no patient achieved a statistically significant change of active or passive dorsiflexion of the ankle angle (with knee flexed or fully extended) at any stimulation frequency.

### Subjective Perceptions of the EXOPULSE Mollii Method

Participants' responses to the semi-structured questions in the questionnaire regarding the self-perception of using the EXOPULSE Mollii suit, are summarized in Table 4. Of note, 15 patients reported feeling of well-being during stimulation at active frequencies and in OFF-settings and five patients a muscle-relaxing effect on the affected hand and/or foot at active frequencies. No participant experienced severe discomfort enough to require the stop of stimulation session, even at the maximum frequency.

**Table 4:** A summary of participants' responses to the questionnaire proposed during the stimulation with EXOPULSE Mollii.

**Open-ended questions**		**Responses**		
**How did you feel putting the EXOPULSE Mollii suit on?**		• No major issue, but perceived need for help to properly put on the suit, due to the poor motor function of the affected hand (*n* = 12)		
		• Increased ease of putting the suit on over time (*n* = 12)		
		**OFF-settings**	**20 Hz**	**30 Hz**
**How do you feel wearing the EXOPULSE Mollii suit?**	**Overall EXOPULSE Mollii suit use**	• Well-being (*n* = 15)		
		• Tight feeling in the body (*n* = 7), perceived as discomfort by one participant		
		• Feeling of warmth, perceived as a positive effect (*n* = 1)		
		• Feeling of cold and stiffness, mainly at the higher frequencies (*n* = 1)		
		• Itching (*n* = 1)		
		Heaviness (*n* = 1)	Slight claustrophobia (*n* = 1)	–
	**EXOPULSE Mollii stimulation**	General sense of stimulation distributed throughout the body, especially in the first minutes of application (*n* = 11)		
		–	• Tingling (*n* = 8)	Tingling (*n* = 8)
			• Muscle-relaxing effect on the affected hand and/or foot (*n* = 5)	
			• Flickering (*n* = 1)	
			• Well-being (*n* = 1)	

Patients' perception of the effort of putting the EXOPULSE Mollii suit on, graded on a 10-point scale, was stable over time: median 3.5 (IQR 3) at day 1 of electrical stimulation, median 3 (IQR 3) at day 2 and 3. Two patients perceived discomfort on one occasion during the hour of stimulation, one patient in OFF-settings (score = 4 out of 10 due to a tight feeling in the body at day 2 of stimulation) and one patient at 30 Hz (score = 1, again at day 2).

Finally, the adherence to the intervention protocol was 100% (i.e., all participants completed the 60 min of stimulation on all 3 days). Of note, 55% of the patients required help of a physiotherapist or caregiver to properly put the EXOPULSE Mollii suit on.

## Discussion

The present study investigated the instant effects of electrical stimulation delivered through the EXOPULSE Mollii suit on spasticity, evaluated with the NeuroFlexor method, EMG, and clinical measures. No significant change in NeuroFlexor NC or EMG amplitude was detected after one session of 60 min stimulation at different frequencies (20 and 30 Hz) and compared with OFF-settings (placebo). Nevertheless, the EXOPULSE Mollii effect was highly variable at the individual level, with reduction in NC especially found among participants with severe spasticity. The subjective perceptions of the intervention were also studied, and the majority of the participants reported feeling of overall well-being during stimulation.

### Evaluation of the Application of the EXOPULSE Mollii Method Every Second Day

The analysis of the potential fluctuations in spasticity over time did not show any statistically significant difference in NC and NeuroFlexor total resistance before stimulation between day 1, 2, and 3, neither in the upper or lower extremity. These results suggest stable spasticity over days and the lack of any carry-over effect of EXOPULSE Mollii stimulation between time points, a prerequisite for the study of changes in spasticity during and after each session of treatment. These results are in agreement with the short-lasting duration of the effects of a standardized application of EXOPULSE Mollii (60 min/session every second day) of up to 48 h, as stated by the company and similarly to the transitory effects of TENS (13).

### EXOPULSE Mollii Stimulation-Associated Changes in Spasticity and Mechanical Contributions to The Muscle Resistance

At group level, no instant positive effect in spasticity was found during or after 60 min of electrical stimulation with the EXOPULSE Mollii suit. Moreover, there were no significant differences between placebo (OFF-settings) and active frequencies (20 and 30 Hz) of stimulation. Although previous findings suggested immediate positive effect of TENS on spasticity (29–31), here both NC and EMG amplitude showed a slight increasing trend after the stimulation with EXOPULSE Mollii at both the active frequencies and even when stimulation was kept off (i.e., OFF-settings), as shown in Figures 4A,B. The static sitting position maintained by the patients during the hour of treatment might explain this slight increase, as suggested by a systematic review of the literature (9) which underlines the importance of activity combined with TENS to treat spasticity. The high variability in degree of spasticity of this study population (e.g., NC before stimulation at day 1 ranged from 3.96 to 56.21 N) might also be a reason of this increase, which was anyway non-significant at group level. Furthermore, it is important to note that the stimulation frequency anticipated by EXOPULSE Mollii differs substantially from the high frequency TENS (i.e., circa 100 Hz) applied in the abovementioned studies. A general tendency of increase after the hour of stimulation was observed even for the NeuroFlexor total resistance and EC, most likely due again to the static sitting position of the patients during the whole session. A new dynamic protocol of treatment can be further developed, including light activities or walking during stimulation with EXOPULSE Mollii.

The number of sessions and the duration of stimulation have been suggested to be key factors in the effectiveness of the treatment with TENS (13, 32). Accordingly, a single session of stimulation with EXOPULSE Mollii might not be a sufficient stimulus to relieve spasticity. Previous studies investigating the effects of repeated applications of EXOPULSE Mollii in adult with chronic stroke (19) (60 min, every second day, for 6 weeks, 21 total sessions) and in children and young adults with cerebral palsy (18) (60 min, 3–4 days per week, for 24 weeks), reported a significant reduction of spasticity, and seem thus to support this hypothesis. It is therefore crucially important that future studies consider this potential dose response and involve multiple sessions of treatment to properly detect the EXOPULSE Mollii effect on spasticity, and to compare the different frequencies of stimulation.

Theoretically, the mechanism of EXOPULSE Mollii in the reduction of spasticity is mediated, similar to TENS (10, 33, 34), by the reciprocal inhibition involving Ia inhibitory interneurons in the spinal cord. However, the stimulation of large diameter afferent nerve fibers by TENS can also modulate neuronal synaptic reorganization (35) and enhance presynaptic inhibition (36), and in addition can lead to transient facilitatory changes in corticomotoneuronal excitability (36, 37). The same mechanisms might be hypothesized to have an adjunctive role in spasticity reduction in the stimulation with EXOPULSE Mollii. One should also consider the potential proprioceptive facilitation of the tight-fitting suit, even when stimulation is kept off (i.e., OFF-settings) (38, 39). Sensory impairments following stroke may additionally impact the individual response to the stimulation with EXOPULSE Mollii. Unfortunately, only a limited number of patients in the current study had intact sensory pathways (*n* = 3 and 2 in the upper and lower extremities, respectively), complicating a proper analysis of the sensory impairment contribution.

**Figure 5:**
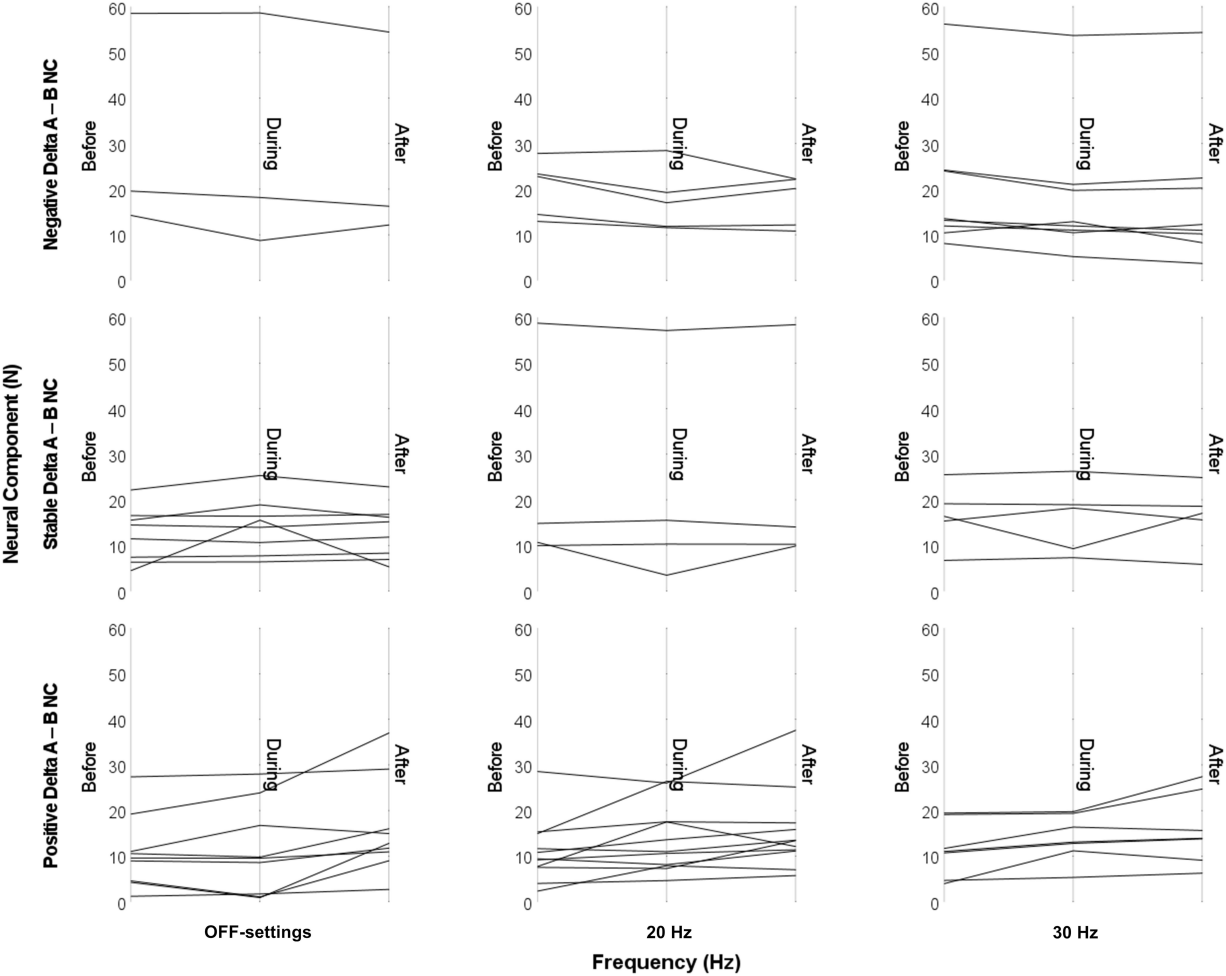
Individual changes in NeuroFlexor neural component in the upper extremity during and after stimulation with EXOPULSE Mollii, graphically presented by type of response (Delta_A−B_ NC). A high variability of the EXOPULSE Mollii effect on NeuroFlexor neural component (NC, in Newton, N) was observed at individual level. Three patients out of 20 presented a negative Delta_A−B_ NC (i.e., the difference in NC between after stimulation value and before stimulation value) in OFF-settings, six patients at 20 Hz and eight at 30 Hz. To notice, NC before electrical stimulation was >14 N for OFF-settings, >13 N for 20 Hz and >8 N for 30 Hz among these patients. Nine patients presented a positive Delta_A−B_ NC in OFF-settings, 11 patients at 20 Hz and seven patients at 30 Hz. Delta_A−B_ NC within the margin of measurement error of 1 N was considered stable.

**Figure 6:**
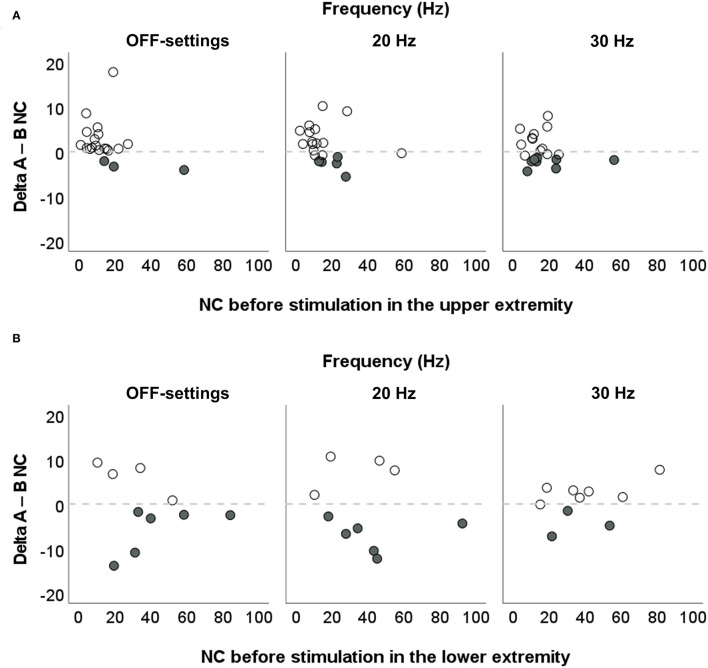
Individual changes in spasticity in the upper and lower extremities after stimulation with EXOPULSE Mollii. Correlation between values of NeuroFlexor neural component (NC, in Newton, N) before electrical stimulation with the EXOPULSE Mollii suit, and Delta_A−B_ NC (i.e., the difference in NC between after stimulation value and before stimulation value) in **(A)** the upper extremity (*N* = 20) and **(B)** lower extremity (*n* = 10). Solid circles represent the reduction in NC beyond the margin of measurement error (i.e., 1 N).

At individual level, the EXOPULSE Mollii effect was highly variable between patients as shown in Figure 5, and even between upper and lower extremities where six patients out of 10 exhibited a reduced NC after stimulation compared with before in OFF-settings and 20 Hz, and three patients at 30 Hz (data not shown). It is important to note that all the patients with decreased NC after the intervention (i.e., negative Delta_A−B_, in Figure 6), presented severe upper limb spasticity with NC > 8 N (5) before the stimulation. Similarly, the previous study (19) showed that NC significantly decreased in the upper extremity after 6 weeks of repeated stimulation sessions and that NC before the intervention could explain 60% of the variance in the difference in the NC of the wrist flexor. Thus, higher NC before the intervention was associated with a greater effect of the stimulation with EXOPULSE Mollii. Evidence from these studies suggests therefore that the EXOPULSE Mollii method might be more effective at improving severe spasticity, with significant implications for clinical practice and recommendations for future research.

The low number of participants properly evaluated with the NeuroFlexor foot module (subsample *n* = 10) represents a main limitation of this study. Moreover, a *posteriori* evidence (i.e., the plausible dose–effect model of the stimulation with EXOPULSE Mollii on spasticity) suggests that a higher number of stimulation sessions could have allowed a more reliable analysis of the effectiveness of the EXOPULSE Mollii suit, an element worth investigation in future studies.

### Subjective Perceptions of the EXOPULSE Mollii Method

As in the previous study (19), the adherence to the study protocol was high (100%) and no severe negative effect or high discomfort was reported by the participants. In accordance with previous findings (19, 40), the difficulty in putting on the suit represented one of the main issues in the treatment with EXOPULSE Mollii, with the effort in wearing the suit rated moderate (3.5 in median out of 10 at day 1, and 3.0 at day 2 and 3). The tightness of the suit, while it allows the electrical stimulation through electrodes integrated in the garment and without the application of gel, might be a limitation of EXOPULSE Mollii method that should be addressed by the manufacturer in view of the next generation of the suit.

To conclude, 75% of participants felt generally well-during stimulation at all frequencies and five patients reported a muscle-relaxing effect on the affected hand and/or foot already within the first 30 min of stimulation at 20 Hz, similarly to the TENS effect on muscle tightness (30).

### Study Limitations

In addition to the above-mentioned limitations related to sample size and number of stimulation sessions, lack of information about individual stimulation parameters limits the interpretation and replication of the current study. This information was considered as “confidential corporate information” by the manufacturer. Thus, while this study demonstrates that the current application of the treatment method may impact on spasticity after stroke, data do not allow any conclusions on how to potentially optimize stimulation variables with respect to an instant effect on spasticity.

## Conclusion

EXOPULSE Mollii is an innovative approach for non–invasive and self–administered electrical stimulation designed to reduce spasticity, a common complication in patients with neurological disorders, including stroke. The 60 min of stimulation with EXOPULSE Mollii at different frequencies (20 and 30 Hz) was not associated with an instant significant reduction of objective signs of spasticity at the group level in patients with chronic stroke. However, the inter-individual variability in response to the EXOPULSE Mollii stimulation was high, especially in participants with severe spasticity. Evidence from this study suggests the need for further studies with higher number of stimulation sessions with EXOPULSE Mollii for patients with severe spasticity after stroke.

## Data Availability Statement

The original contributions presented in the study are included in the article/Supplementary Material, further inquiries can be directed to the corresponding author/s.

## Ethics Statement

The studies involving human participants were reviewed and approved by Swedish Ethical Review Authority (2017/935-31). The patients/participants provided their written informed consent to participate in this study.

## Author Contributions

JB and SP have made substantial contributions to the conception of the work. GP, JB, and SP to the design of the work. GP and HB performed the data collection. GP the MATLAB analysis in collaboration with LC. GP, PL, JB, and SP contributed to the statistical analysis and interpretation of data. GP wrote the manuscript together with PL, JB, and SP. All authors approved the submitted version.

## Funding

This study was funded by grants from Eurostars, EUREKA, and the European Commission (https://www.eurostars-eureka.eu), reference: 10627/15/24722/Ae, and the Promobilia Foundation. The EXOPULSE Mollii suits were provided by EXONEURAL NETWORK AB. The choice of the stimulation parameters was performed by a member of the manufacturer and guided by the clinical practice. The study sponsors had no other role in study design, data collection and analysis, interpretation of results, preparation of the manuscript, and/or the decision to publish.

## Conflict of Interest

The NeuroFlexor instrument has been patented (WO/2008/121067), and the PL is a shareholder in the manufacturing company Aggero MedTech AB. The remaining authors declare that the research was conducted in the absence of any commercial or financial relationships that could be construed as a potential conflict of interest.

## Publisher's Note

All claims expressed in this article are solely those of the authors and do not necessarily represent those of their affiliated organizations, or those of the publisher, the editors and the reviewers. Any product that may be evaluated in this article, or claim that may be made by its manufacturer, is not guaranteed or endorsed by the publisher.

## Abbreviations

EC: elastic component
EMG: electromyography
FCR: flexor carpi radialis muscle
FMA–LE: Fugl–Meyer scale for lower extremity
FMA–UE: Fugl–Meyer scale for upper extremity
IQR: interquartile range
MAS: modified Ashworth scale
N: Newton
NC: neural component
ROM: range of motion
TENS: transcutaneous electrical nerve stimulation
SD: standard deviation
VC: viscous component.
