# Supplementary material for: Effects of 60 Min Electrostimulation With the EXOPULSE Mollii Suit on Objective Signs of Spasticity
Source: Front Neurol. 2021 Oct 15;12:706610. doi: 10.3389/fneur.2021.706610 (PMC8554021; doi:10.3389/fneur.2021.706610)
Supplement: Supplementary file 1 [file Data_Sheet_1.PDF]

Supplementary Material

Supplementary Material

SUPPLEMENTARY FIGURE

Figure 1. Flow chart showing cross-over design and assessment points. NeuroFlexor neural component (NC), in Newton, N.

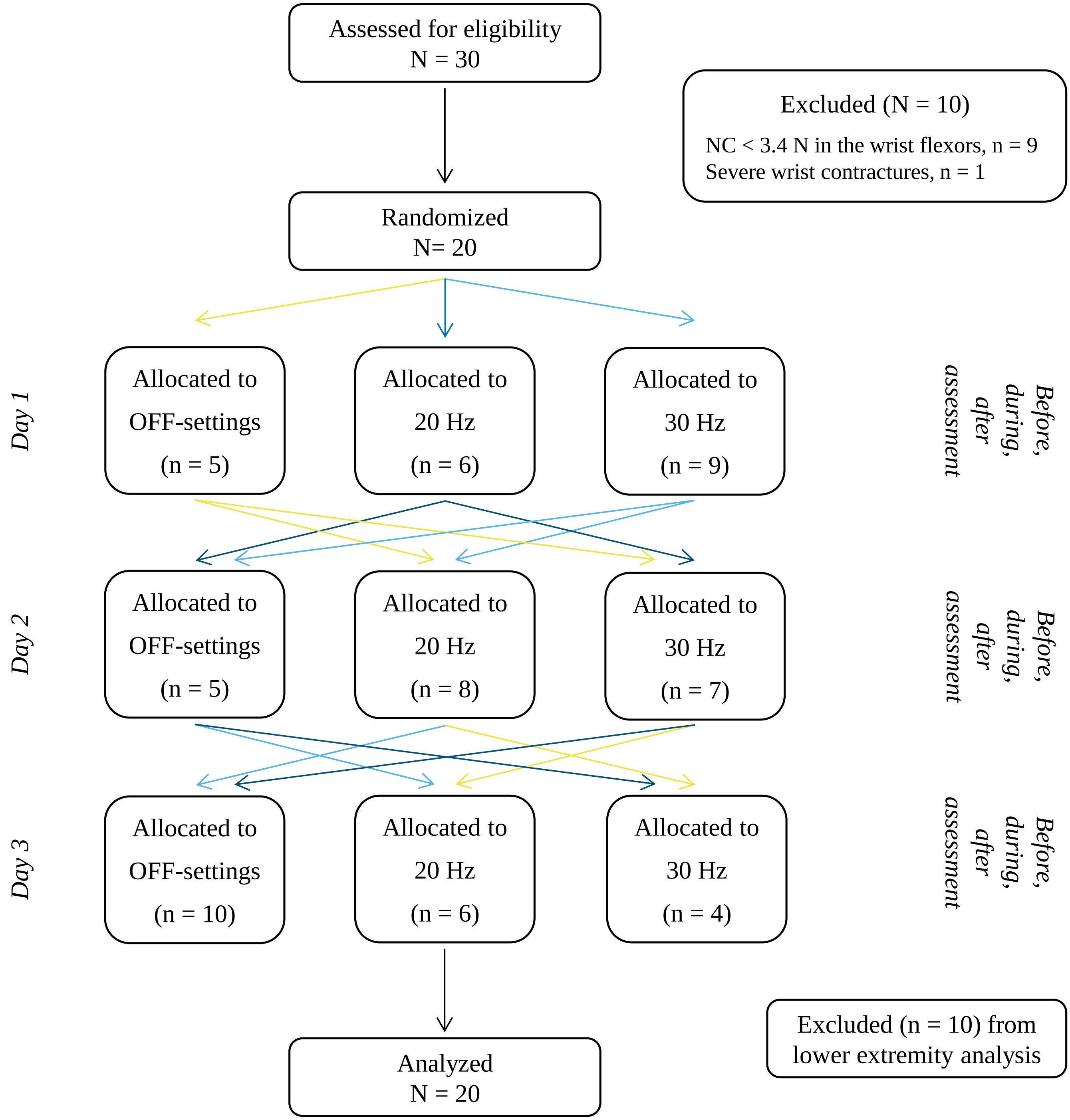

SUPPLEMENTARY MATERIALS AND METHODS

The NeuroFlexor model

The biomechanical model of the NeuroFlexor, previously described by Lindberg et al.(1), analyzes the resistive force resulting from passive wrist extension or dorsiflexion of the ankle at two velocities, and distinguishes the different contributions: inertia, elasticity, viscosity, and the neural component.

The inertia is the force resisting the acceleration of the hand or the foot.
The elastic component (EC) is a length-dependent resisting force to the stretch, and it is recorded 1 second after the end of the slow movement (at 5°/ s).
The viscous component (VC) is a velocity-dependent resisting force produced by friction from neighboring tissues, for example sliding muscle fibers. VC is highest during the initial acceleration and continues at a lower level (approximately 20% of the early viscosity) during the fast movement at 236°/s for the upper extremity and 240°/s for the lower extremity.
Finally, the neural component (NC) is the stretch reflexes mediated force (i.e., spasticity) and it is estimated in the model at the maximal stretch by subtracting EC and VC from the total resistance.

Data on validity, reliability and sensitivity to change of the NeuroFlexor method have been described (1-3), and cut-off values for the passive and active contributions to increased upper limb muscle tone were defined (4).

SUPPLEMENTARY REFERENCE

1. Lindberg PG, Gaverth J, Islam M, Fagergren A, Borg J, Forssberg H. Validation of a New Biomechanical Model to Measure Muscle Tone in Spastic Muscles. *Neurorehabil Neural Repair*. 2011;25(7):617-25.

2. Gaverth J, Sandgren M, Lindberg PG, Forssberg H, Eliasson AC. Test-retest and inter-rater reliability of a method to measure wrist and finger spasticity. *Journal of rehabilitation medicine*. 2013;45(7):630-6.

3. Gaverth J, Eliasson AC, Kullander K, Borg J, Lindberg PG, Forssberg H. Sensitivity of the NeuroFlexor method to measure change in spasticity after treatment with botulinum toxin A in wrist and finger muscles. *Journal of rehabilitation medicine*. 2014;46(7):629-34.

4. Pennati GV, Plantin J, Borg J, Lindberg PG. Normative NeuroFlexor data for detection of spasticity after stroke: a cross-sectional study. *J Neuroeng Rehabil*. 2016;13:30.
